# Creativity across domains: Thoughts in science, engineering, mathematics, computer science, technology, and art

**DOI:** 10.1093/pnasnexus/pgaf174

**Published:** 2025-07-01

**Authors:** Julio M Ottino

**Affiliations:** Ford Engineering Design Center, Northwestern University, 2133 Sheridan Road, Evanston, IL 60208, USA; Robert R. McCormick School of Engineering and Applied Science, Kellogg School of Management, Northwestern University, Evanston, IL 60208-3100, USA

## Abstract

Astounding examples of creativity abound in science, engineering, mathematics, computer science, technology, and art; in fact, creativity is essential to their functioning and growth. Manifestations that cross, blur, link, and synergize these domains have resulted in concepts and ideas that make us proud to be human. Much has been written about creativity, but studies are unevenly represented across domains. This perspective will touch on all the previously mentioned domains, span individuals and teams, and intertwine archetypal historical examples of creative fluidity with how creativity may be affected and accelerated by computational tools and artificial intelligence. The objective of this piece is to present a broad and unified perspective of what is a vast creativity landscape, a view that may be lost when focusing on components rather than the whole.

## Introduction

“How something comes out of nothing.” This could be a perfect description of thinking in pure mathematics, but it is the subtitle of *The Work of Art*, a recent book that focuses on creative processes and how outputs emerge in various artistic fields (1). While books on artistic areas are common, the number of accounts of creative processes underlying physical (2), mathematical (3), and engineering thinking (4) is, compared with those covering art, exceedingly small. In fact, the number of first-person accounts of creative thinking coming from physical sciences and engineering is relatively rare. It is far more common to see studies and accounts of creativity in psychology/management (5, 6), and even economists studying art (7). In this paper, I will not try to survey the whole literature on creativity. In fact, I will be leaving out significant aspects of human creativity, like writing and music. I will neither get into aspects of the psychology and models of creativity (8–10)—topics that may force us to define terms like *intuition*, *inspiration*, and *insight* (11, 12)—nor survey neuroscience studies exploring the root causes of creativity (13). Finally, I will not attempt to define creativity itself (14) or articulate a theory of creativity. However, I will describe different classes of creativity, propose a benchmark for the highest level of creativity—something I call a “break-with”—and provide a perspective on the potential for computers to produce creativity. Implicit in the coverage is the belief that creative fluidity is a good thing, and I will give examples of combining modes of thinking that have led to greater creativity.

## The landscape: Stereotypes, perceptions, and terms

Let me start by acknowledging that discussions of creativity may take place amid persistent stereotypes and terms that have evolved in the course of time. Here, I cover 3 of them.

The first is that there is a strong tendency to draw sharp distinctions between art and science. The German philosopher Immanuel Kant (1724–1804), for whom not even Issac Newton qualified as creative, argued that “science is ephemeral, art is permanent.” The view from some quarters of the humanities is equally clear if myopic: “Science may appear creative only because we forget what creativity really means and take it to be cleverness at proposing hypothesis, finding proofs or inventing experiments” (15). This is especially reductive when pronounced after the advent of quantum mechanics and relativity. The root of the argument is that artistic creations are personal. Without T.S. Eliot, we would not have *The Waste Land*; on the other hand, the argument goes, if Thomas Edison had not lived, someone would surely have stumbled into the light bulb. Newton's laws would have been discovered inevitably, though possibly in a different form, by someone else.

Associating creativity with the arts, and idea entrenched by the Romantic Era (16), is a persistent and commonly held stereotype. It persists even with domains themselves. Most scientists and engineers equate art with creation, beauty, inspiration, and sometimes struggle, and they envision art as paintings, photographs, and sculptures, leaving out conceptual art, installations, and much more. Most artists equate science with meticulous and dispassionate thinking and engineering with cold technology, rationality, and practicality, and not with the human factors and passions that animate them.

A second persistent point is the continual, almost irritating reference, to C.P. Snow's *The Two Cultures* as a point of departure in the popular press in seemingly any account of science and an art. In that speech, then published as book (17), Snow posited a split between the worlds of “science” and “the humanities” in the British educational system, which, in his view, had overemphasized the humanities (especially Latin and Greek) at the expense of scientific and engineering education. It is important to note that the “humanities” Snow referred to was mostly writing.

And finally, the meanings of “art,” “technology,” and “science” have changed historically over time. Until the 19th century, “arts” or “useful arts” was often a synonym for what was later named technology, and the distinction between “art” and “technology” was not clear until then. That is how art appears in the National Academy of Sciences chartering document: “[The] Academy shall, whenever called upon by any department of the Government, investigate, examine, experiment, and report upon any subject of science or art.” Humanity has been doing technology, and displaying creativity, long before engineering became an established discipline—think, for example, of the Bronze Age and Roman aqueducts. But the word *technology* itself is relatively recent, arguably used for the first time in the very early 20th century (18).

Terms, though often incomplete, have the nasty habit of resisting redefinitions. Let us consider a processes/outcomes framework, so that we might be able to see both distinctions and overlaps.

Science and engineering are processes. So is art. One could argue that the outcome of science is more knowledge (and more science), that the outcome of engineering is technology, and that the outcome of art is art (*artworks* sounding a bit prosaic). Confusingly, some terms appear as both processes and outcomes. Adding “design” into the mix increases the complexity. The outcome of design is products, but it may also be technology, and the outcome of technology could be products, but it is also technology. As for engineering and technology, let me restate that not all technology emerges from engineering and not all engineering thinking results in technology. It is an understandable desire to clean up this complicated Venn diagram of words and meanings. However, the effort involved in battling and restructuring history should be accompanied by a comparable or greater advance in future clarity in understanding.

It is apparent, however, that within this set, the art and science pair is the one that generates widest interest among the public. A recent Getty exhibit, *PST: Art & Science Collide*, generated coverage in the *Financial Times*, *The New York Times*, *Science*, and *The Wall Street Journal*. Something in the topic (and the invariable reference to C.P. Snow) seemed to strike a chord with journalists, and some pieces went into historical description of how artists viewed science. This suggests that we start back in time, in the Renaissance in Europe, a period that generated astounding examples of art-science-technology collaboration. Now it may be a good point to state that in all of the examples of art, the accent will be on visual art.

## The history: Canonical illustrations of creativity fluidity

In what follows, I present a few historical and biographical examples of what I call creative fluidity: the ability to cross and blur domains.

### Filippo Brunelleschi's engineering prowess: Florence's daring display of confidence

In 1418, those responsible for building the cathedral of Florence held a competition. Entrants needed to find a way to enclose the huge space on top of the cathedral that was left open by the previous generation of planners and builders. The Florentines had been so self-confident and ambitious that they had permitted the cathedral to grow with no clear plan or technological means to complete it. The builders wanted to top it with a dome, which would be a major engineering feat. The dome of the Pantheon in Rome, built almost 1,500 years earlier, was the only other example of such engineering. Since then, no one had produced anything close to a dome of that span (parenthetically, we know who commissioned the Pantheon but do not know who the architect/engineer was). Remarkably, Florence's rulers believed that someone in its midst could tackle and solve the problem. A competition was announced, and 2 finalists emerged: Lorenzo Ghiberti (1378–1455) and Filippo Brunelleschi (1377–1446). Both had been trained as goldsmiths.

After Brunelleschi won and received the commission, he had to overcome aesthetic and engineering problems and also had to invent and construct machines for transporting and maneuvering materials (19, 20). In all of these realms, he was successful. His design was the first octagonal dome in history built without a temporary wooden supporting frame (21). It is still the largest brick and mortar dome in the world. Unfortunately, no plans detailing the construction survive, but it can be argued that Brunelleschi was the first modern engineer (22).

Brunelleschi typifies Florence's creative fluidity: he played transformative roles in the fields of engineering, sculpture, and painting, and through perspective and geometry contributed to key areas in what we today think of as science.

### Galileo's Moon craters and sunspots

Galileo Galilei (1564–1642) and the Englishman Thomas Harriot (1560–1621) are at the root of one of the best examples of the interplay between art, technology, and science (23).

Perspective tubes, or what we now would call a telescope, had been developed at the time for military uses, like spotting invading ships. No one had used them to observe astronomical phenomena. Perhaps that was because many Europeans in the Middle Ages thought that the Moon did not need to be studied. It was a perfect sphere, many believed, and it represented purity; the Virgin Mary was often represented standing on it. In 1609, both Harriot and Galileo observed the Moon with perspective tubes. Both recorded their observations, though Harriot made ink drawings while Galileo painted with watercolors. Galileo also had another skill set: Euclidian geometry.

Galileo spent a significant portion of his life in Florence, where his family was originally from, and considered himself connected to the Florentine community. This teaching was encapsulated in Florence's prestigious *Accademia delle Arti del Disegno*, where a professional mathematician taught elements of Euclidian geometry to aspiring artists. Galileo mastered the teachings of the *Accademia.* This helped him understand shadows projected by complicated objects, which was of immediate help in interpreting the results of his observations. The Moon was not a perfect sphere; changing shadows revealed a surface pocked with craters. Galileo even had the tools to estimate the craters' heights. Harriot had no such observations.

An almost repeat event took place in the summer of 1610, when Galileo and Harriot both observed the Sun's sunspots by means of an improved telescope. Galileo noticed that as the spots approached the edge of the Sun's disk, they became narrower and slower. That movement was consistent with spots on the surface of a rotating sphere (24). He concluded that the sun rotated on a fixed axis and that that sunspots were features on the surface of the Sun. Incidentally, the same year, Galileo, using a telescope of his own design, discovered 4 satellites orbiting Jupiter.

Florence gave Galileo the edge; Harriot who was in London, then far behind Florence. Galileo's visual imagination and training in geometry and perspective allowed him to interpret his findings correctly.

### Santiago Ramón y Cajal: Art illuminating science

Santiago Ramón y Cajal (1852–1934) is regarded as the father of modern neuroscience, but his success stemmed from the fact that he was also a talented artist who used drawing as a form of thinking (25). He (along with Camillo Golgi) received the 1906 Nobel Prize in Medicine “in recognition of their work on the structure of the nervous system.” Cajal's experiments untangled the basics of neuroscience, showing that the nervous system is made up of millions of separate nerve cells called neurons. He discovered that the relationship between neurons did not form a single system, as proposed by others, but was rather contiguous, with gaps between neurons. These insights could be attributed to his artistic eye, for he was truly as much an artist as a scientist. He was a rebellious teenager who had decided to become an artist, then eventually moved into medicine. Still, his artistic talent proved important. His drawings are not exact reproductions of what he saw under the microscope. He drew freehand, combining insights from multiple observations in a single drawing, encapsulating his hypothesis about brain connectivity. His 1906 Nobel Prize Lecture, “The Structure and Connexions of Neurons,” includes 23 figures. Those figures mattered. Ramón y Cajal's meticulously detailed drawings were crucial in settling the conflict between Camillo Golgi's reticular theory of the nervous system and Ramón y Cajal's neuron doctrine [details are given in Fokas (26)]. Many of Ramón y Cajal's more than 3,000 drawings are still in use today, helping many others extract insights from the images.

### Louis Pasteur: The discovery of chirality, inspired by art?

Louis Pasteur (1822–1895) made numerous contributions to science, technology, and medicine. His mode of work, which merged basic and applied research, is now encapsulated in the concept of the Pasteur's Quadrant (27), the space where curiosity-driven and application-driven research coexist.

Pasteur was passionate about art (28). In his teens, he was an average student but an accomplished artist, creating dozens of portraits of family and friends using pastels, charcoals, and significantly, lithographs—an art form that serves as training to see images and their mirror images. At age 20, Pasteur shifted his focus to science, but he continued drawing figures for his scientific publications, including representations of crystals and images for his microbiological work. As his career progressed, Pasteur remained intensely interested in the arts; he attended salons, kept a diary of art that he saw at museums, and taught classes at the École des Beaux-Arts on how chemistry could be used in fine art.

After graduating from the École Normale Supérieure, he began to study optically active substances—substances that rotate polarized light (29, 30). In 1848, shortly after receiving his doctorate, he made a surprising observation. What seemed like an undistinguishable mixture of crystals was, in fact, a mix of two kinds of crystals: a 50–50 mix of two nonsuperposable mirror-image crystal forms, chiral images of each other (31). Pasteur had discovered chirality at age 25. It was incredible that he could have discerned this pattern. Imagine a heap of crystals and somehow discovering that, when orienting two of them properly, they happen to be mirror images of each other. Pasteur left no notes of his thought process, but one could argue that his work dealing with mirror images in lithography was essential to this discovery.

### From Poincaré to Picasso and Duchamp: Mathematics influencing art

Jules Henri Poincaré (1854–1912) was often referred to by his contemporaries as the last complete mathematician, the “last of the universalists.” Unusual for a mathematician, Poincaré was an essayist who wrote about the psychology of creativity and even explained the role of intuition and the subconscious in his thinking process. He was so gifted in both writing and math that he was elected to the French Academy for his literary achievements and also served as president of France's Academy of Sciences. His book *La science et l’hypothèse* (*Science and Hypothesis*) was required reading for any cultured person, especially in France.

In fact, Poincaré's scientific ideas influenced artists at the time. Esprit Jouffret (1837–1904), a French artillery officer, actuary, and mathematician, popularized Poincaré's in a book titled *Traité élémentaire de géométrie à quatre dimensions* (*Elementary Treatise on the Geometry in Four Dimensions*), published in 1903. Then, Maurice Princet (1875–1973), known as “le mathématicien du cubism,” brought Jouffret's work, most notably the concept of the “fourth dimension”—an extension to our everyday perception of the 3-dimensional space—to two influential artists: Pablo Picasso (1881–1973) and Marcel Duchamp (1887–1968). Picasso’s sketchbooks for *Les Demoiselles d'Avignon*, generally referred to as the first Cubist picture, illustrate Jouffret’s influence. Duchamp's case is even clearer: He left notes referring to Jouffret's book (32).

### Niels Bohr: Using cubism to explain quantum mechanics

**Fig. 1.**
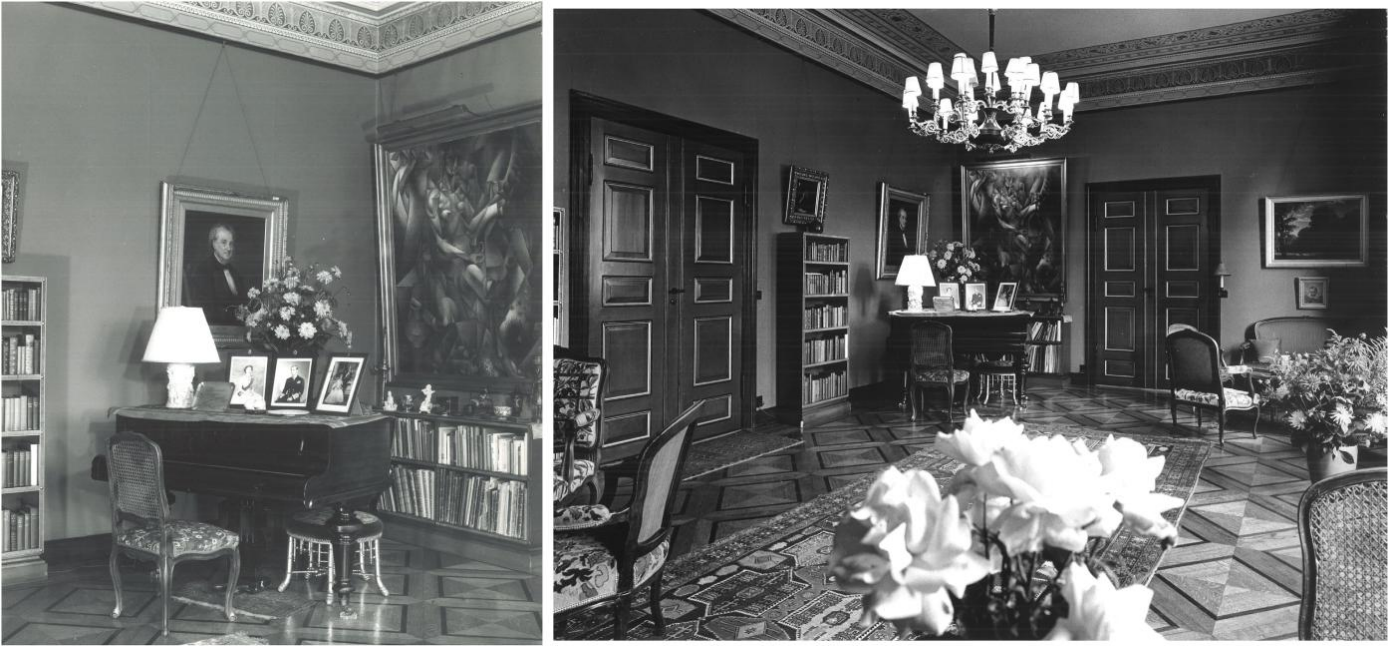
Niels Bohr’s house in Copenhagen, now the Carlsberg Academy. The large painting on top of bookcase in the left image is Jean Metzinger’s *La Femme au Cheval.* Bohr used this cubist work to explain the idea of complementarity to visitors. The right image captures the drawing room itself where Niels and Margrethe Bohr could host visitors in style. Photos courtesy of the Niels Bohr Archive.

Physicist Niels Bohr helped develop quantum mechanics in the early 20th century, conceiving of the idea of complementarity: a far from intuitive concept that something can be two things at once. In 1921, with the help of the Carlsberg Foundation and the Danish government, Bohr founded the Institute for Theoretical Physics in Copenhagen. After Bohr won the Nobel Prize in 1922, the Carlsberg Foundation gave him a house where he could host visitors. Bohr enjoyed cubism and modern art, and art was a centerpiece of this home; “Several twentieth century paintings in more modern style hung in various places” (33). One of those artworks was the 1911 cubist painting *La Femme au Cheval* by Jean Metzinger (see Figure 1). Bohr's relationship with this painting is well documented. Mogens Andersen, a friend of Bohr's eldest son, himself a painter, wrote a short piece titled “An Impression” for a book edited by Stefan Rozental (34), who was Bohr's assistant for 15 years. In this book, Mogens explained Bohr's relationship with *La Femme au Cheval*. “I have often seen Niels Bohr, full of life, explain his views and interpretation of the picture. In his eyes there was the pleasure of giving form to thoughts to an audience unable to see anything in the painting.” Bohr used this cubist work to explain the idea of complementarity. “Bohr was engrossed with the ambiguity of the motif … that an object could be several things,” Mogens wrote.

All these examples indicate the ways that creative fluidity has produced inspiring and remarkable outcomes. I will focus now on how the different domains work.

## Creativity as an interaction between individuals, domains, and fields

An enduring myth about creativity is that ideas appear out of nowhere. In fact, creative ideas do not happen in a vacuum. Creativity is not a private enterprise but involves the interaction of 3 things: (i) a person or a group; (ii) a domain, the branches of mathematics or physics or visual arts or history, for example; and (iii) the field, the set of people who are gatekeepers of the domain (35).

Let us define these terms further. Art, technology, science, and mathematics are domains, and so are components of the domains (like condensed matter physics, cultural anthropology, and Renaissance history, for example). A domain includes the accumulated recorded knowledge and the implicit rules that govern the addition of new knowledge into the domain itself (theories, models, methodologies, and principles).

Each domain works differently. Science builds on the past; it is about methodically building and adding knowledge (36). Incremental progress is the path forward; massive revolutions are rare. The objective is knowledge. Technology, on the other hand, is always about building and disrupting. The objective is building, and disruption is necessary for growth.

And art, especially as we move into the 20th and 21st centuries, is seemingly all about disruption. Modern art's aspiration is uniqueness; disruption and progress has little or no meaning. Art does not solve problems; it creates questions.

Who decides what stays in the domains? The field. Fields make collective decisions via gatekeepers (for example, article referees in sciences; curators, critics, museums, and collectors in art) as to what becomes part of the domain. That is, what goes in, what stays out, and even what may get rediscovered.

The idea of progress and innovation is central to creativity, and this is how some fields decide what is new and what should go in. But the idea of innovation as something good is, in terms of human history, a rather recent concept. Before the Renaissance, people believed that knowledge resided in the past. Book hunters searched for traces of that knowledge in manuscripts preserved in monasteries. One such semi-popular account is described in the book *The Swerve* (37). One can argue that the root of modernity is when people started seeing knowledge as cumulative, and the idea of progress as something positive (38).

Progress is sometimes encapsulated in stories, but caution is in order. Storytelling has a way of solidifying some of those myths and stereotypes that keep us from understanding the real conditions of creative fluidity.

## Creative fluidity, real and imagined

In fact, it can be easy to overstate creative synergies. In his book *Einstein, Picasso: Space, Time and the Beauty that Causes Havoc*, Arthur Miller (39) writes, “If cubism is the result of the science in art, the quantum theory is the result of art in science.” This is a nice, symmetric argument but probably an oversimplification. It is nevertheless hard to imagine another time in which the foundations of science and art changed in parallel at such rapid pace. It is undoubtedly true that interconnections existed, and that both art and science together changed forever.

A few years ago, I checked with the archivists at the Niels Bohr Institute to see if Niels Bohr had Metzinger's and Albert Gleizes's book, *Du Cubisme.* The answer was no. We do know for sure that Bohr used cubism to explain complementarity. That was the key point of the vignette. But the finding could have transformed the story into a myth.

It is indeed easy to romanticize narratives about intersections. In fact, the same as with celebrated quotes, in which time has constructed phrases that historical persons could have said but did not (about half of celebrated quotes out there do not withstand scholarly scrutiny), we may endow famous scientists with skills that they did not possess.

Jacobus Henricus van’t Hoff (1852–1911) was the first winner of the Nobel Prize in Chemistry and is widely considered as one of the founders of physical chemistry. In 1960, the *Journal of Chemical Education* reprinted van’t Hoff's inaugural speech as professor of chemistry at the University of Amsterdam. The title of his presentation was “The Role of Imagination in Science” (40). A note accompanying van’t Hoff's piece comments that this was the second article in the *Journal of Chemical Education* focusing on scientific creativity, the first being by August Kekulé. Kekulé's account of solving the structure benzene is famous. As he laid by a fireplace, looking at flames, he had a vision of a snake devouring its own tail. That led him to realize that the carbon atoms of benzene must be organized in hexagon.

Van’t Hoff's lecture is one of the rather rare cases of a scientist describing when an idea occurred to them. He offered suggestions about the value of the subconscious in research. “I will give you recipe for making discoveries … [libraries] have always had a deadening influence on my mind.” He said that while he was engaged in studying an article at the Utrecht library, “I interrupted my perusal halfway in order to go for a walk. It was on this walk that through the influence of the fresh air the idea of the asymmetric carbon atom arose in me.” Henri Poincaré has a similar story: after taking a walk and stepping into his bus, the solution to a problem he had been working on suddenly appeared fully formed. His subconscious mind had been putting the pieces together. Same with van’t Hoff.

Van’t Hoff manages to connect his example to “whether this capacity [in scientists] revealed itself also in ways other than in their scientific research.” He tells us that “On studying more than two hundred biographies, it turned out that this was in fact the case and to a very large extent.” He then goes on to single out “artistic expression of Imagination.” Newton and Galileo are among several names mentioned by van’t Hoff in his lecture. His source is François Arago's *Ouevres Complètes*. François Arago (1786–1853) was an accomplished French physicist who made significant discoveries in, among other areas, magnetism and light polarization. Van’t Hoff, quoting Arago, pairs Newton with “marked success in the art of painting” and Galileo with being “[a] great admirer of Ariosto. Knew all the *Orlando Furioso* by heart…took part in the greatest disputes of his times…on the comparative merits of Ariosto and Tasso.”

There is evidence for the Galileo attribution, but there is no evidence that Newton did any serious painting. It is apparent that the biographical mode of explaining creative inspiration may be flawed. Accounts may not withstand scrutiny. Also, individual stories could be part of the barriers to creative fluidity, because they do not let us see elements of crossing domains.

What prevents creative fluidity in today's world?

## Barriers to creative fluidity in science

Creative fluidity abounded in the Renaissance, but by the time of the Enlightenment, disciplinary boundaries between art and science hardened. What prevents creative fluidity from happening now, even on a much smaller scale that that of the Renaissance? I will single out 2 reasons: the impossibility of mastering all domains and science becoming overly dominated by analysis and rationality. It is hard to overcome the first reason, but there may be some glimpses of hope for the second.

The impossibility to master all domains is encapsulated in the story of Mary Fairfax Somerville, a Scottish polymath. In 1834, she published a book titled *On the Connection of the Physical Sciences* (41), an attempt to summarize all scientific knowledge accumulated up to that time, including physics, astronomy, meteorology, and geography. It was wildly successful and is often regarded as the book that launched popular science writing.

William Whewell, an extraordinary figure in his own right, wrote a 13-page review of Somerville's book. In his review, Whewell could not refer to Somerville as a “man of science,” the common term at the time. He sought a word that would reflect the interdisciplinary nature of Somerville's expertise. Whewell decided to use a word of his own invention: *scientist*, the first use of that term (42). As her book demonstrated, Somerville was more than a mathematician, astronomer, or physicist—she seamlessly blended these areas.

But her book was quite possibly the last chance that anybody could summarize all known science in a bit over 500 pages, as science experienced explosive growth afterward. Maxwell came up with electromagnetism; Maxwell, Boltzmann, and Gibbs developed statistical mechanics and thermodynamics, and Poincaré put an end to determinism with the discovery of chaos. It would subsequently be impossible to cover all known science.

When did science become dominated by rationality? Possibly the best example is Joseph-Louis Lagrange. His *Analytical Mechanics* (1788) became the most influential work on classical mechanics since Newton's *Principia*; it ultimately charted the course of mathematical physics in the following century. In it, Lagrange states, “No pictures will be found in this book.” In his view, pure analytical reasoning needed no aides. It can be difficult to imagine connections with other domains when you see your field as the apex of thinking.

Physics and mathematics have often been portrayed as examples of domains in which images play no role. But this view is far too narrow. Visual imagination, of course, was always there, but is sometimes hidden below the surface. Visual thinking may have helped in the elaboration of ideas but was hidden away in the final presentation of results. The historian of science Gerald Holton wrote on the topic of scientific imagination (43), and so did the mathematician Jacques Hadamard (44). Examples of visual imagination include Albert Einstein (1879–1955) and Richard Feynman (1918–1988). The German physicist Werner Heisenberg (1901–1976) was squarely on the “no” side (“the progress of quantum mechanics has to free itself from all these intuitive pictures”), as was the mathematician Karl Weierstrass (1815–1897).

“You may leaf through all his books without finding a figure,” Poincaré said of Weierstrass (Hadamard 1945). Michael Faraday (1791–1867) was on the visual side, and Bernhard Riemann (1826–1866) was on the nonvisual one. I can add James Clerk Maxwell (1831–1879), especially for his less well-known work in mechanics, as a remarkable example of the visual imagination side (45).

## How artists have changed since the renaissance

Science has changed quite a bit since Lagrange's times. But artists, and the practice of art, has changed as well, perhaps even more. If science has become more rational, then art has become more irrational, that is, more separate from the tradition of physical creation. Painting and sculpting, before the Renaissance, were equated with physical labor, not mental or intellectual labor. In Spain, for example, this view lasted until the first years of the 17th century. It was in the Renaissance that art was equated with creation, and with it, the emergence of apprenticeships. The Romantic period, in the late 18th and early 19th centuries, gave us the cult of the artist breaking rules and overthrowing tradition and the emergence of the term *fine arts* (46). Modernism, the age that gave us Picasso, Virginia Woolf, and Igor Stravinsky, made artists the new cultural aristocrats. After World War II, and in America in particular, MFA programs emerged, and after that, some artists became entrepreneurs, something in full force today.

## How creative ideas emerge

Let us divide creativity into 2 classes that will serve to anchor the discussion when we get to specific examples. The first class can be called combinative creativity—the process of combining existing ideas, concepts, or elements to generate novel solutions.

Most, if not all, creative ideas are combinations of past ideas. Many creative people have recognized this fact. Versions of “*O*riginality is undetected plagiarism” and “Originality is nothing but judicious imitation” can be attributed to the French philosopher Voltaire and to the poet Lord Byron. Einstein is credited, along with others, with “The secret of creativity is knowing how to hide your sources.” Thus, while we may romantically think we can create ideas and concepts out of thin air, what we are doing is mining and building upon the past. Innovations are mostly based on combinations of existing ideas.

Combining two things to create a new third is relatively common. Combining 3 elements to produce something unique is much less so, but the essential combination at the core of many creative advances. The Wright brothers and the invention of the airplane could be imagined as the coming together of the knowledge of gliders, the use of aluminum, and the wind tunnel. Guglielmo Marconi's invention of the radio was the merging the telegraph, telephone, and radio frequency vacuum tube. The first affordable automobile developed by Henry Ford could be seen as combining the maturation of the gasoline engine, petroleum refining, and the concept of the assembly line. This list may even include things like the iPhone: the large-scale integrated circuit, the gallium arsenide radio chip, and the lithium battery.

Many times, separate scientists and engineers have combined 3 ideas at the same time, manifesting in multiple discoveries (47). The telephone emerged from combining 3 key ideas and technologies: an understanding of electrical current and electromagnetic principles, the ability to convert sound waves into electrical signals, and a discovery that a membrane or diaphragm could be made to vibrate in response to sound waves. Who had this idea? Both Alexander Graham Bell and Elisha Gray, who both filed patents in the United States the same day.

James Clerk Maxwell produced what is considered the first demonstration of color photography in 1861 by taking 3 separate black-and-white photographs through red, green, and blue filters and then combining them into a single image. That was one key component in a bigger idea. Eight years later, Charles Cros and Louis Ducos du Hauron combined understanding of color theory, photographic chemistry, and multiple exposure techniques to create color photographs. The concept of the adjacent possible, originally introduced to explain biological evolution by Stuart Kauffman, provides a way of explaining the multiplicity of multiple discoveries (48).

It is also useful to imagine how entire disciplines—not just technologies—may be combinations of 3 components. Modern medicine could be imagined as the combination of information in the form of medical science and knowledge (all the underpinning science, beginning with germ theory and moving into molecular biology, genomics), new classes of machines (x-rays, magnetic resonance imaging, robotics, diagnostics), and new kinds of materials (pharmaceuticals would fall in this bucket, as well as implantable and surgical materials).

A much earlier date for the emergence of the modern era of medicine, having roots on an art/science intersection, should be mentioned as well: the 1543 publication of *De Humani Corporis Fabrica* (*On the Fabric of the Human Body*) by the Flemish neurosurgeon Andrea Vesalius, which contains detailed realistic illustrations created by apprentices of the great painter Titian.

## Beyond the break-through: The break-with

There is also a second class of creativity that involves radical shifts in perspective, leading to the creation of entirely new paradigms or frameworks. It is creativity that is more closely linked to disruptive innovation—what many people refer to as “breakthroughs.” Breakthroughs represent a sudden development and a new insight. The term itself suggests something that pierces the boundary of a domain of knowledge and enlarges the domain.

But there is higher bar: a “break-with.” A break-with is conceptual advance that breaks with ideas that were at the very center of the previous way of thinking. In extreme cases—a paradigm shift, in Thomas Kuhn's language (49)—new domains form, and a new order sets in. This kind of creativity is rare, equivalent to the advent of quantum mechanics in science or cubism in art. One may polish these concepts to recognize hierarchies within breakthroughs and break-withs.

## Understanding modern art as a break-with

For a long while, all visual art was representational. This changed in the early 1900s. Along with the emergence of quantum mechanics and relativity, cubism appeared, and prestige moved away from representational art. What were a series of gradual changes—mannerism replaced by baroque, then rococo, then neoclassical painters—gave rise to a discontinuity. In the early 20th century, in a time span of less than 25 years, the modern movement turned neoclassical academic giants such as William-Adolphe Bouguereau, Ernest Meissonier, and Jean-Léon Gérôme into nearly forgotten figures. After that, there was a quick succession of periods: the modern, postmodern, and post-postmodern may have been the last recognizable ones (50). Peter Schjeldahl, the late art critic for *The New Yorker*, put it in vivid language: “Then those movements, too, disintegrated, and it's been pretty much one damn thing after another ever since.”

It is instructive to compare science with art. Science is making the unfamiliar familiar or making the invisible visible. We could argue that art, especially modern and contemporary art, is the opposite. It is seeing something that we may have seen a hundred times before but now seeing in a different light, a new viewing that makes the familiar become unfamiliar. This is “bestrangement,” as the Russian formalists called it (51), or “perplextion.” Sometimes described as the moment of awe, it is the first step toward a critical engagement with art. These comparisons can extend to engineering and humanities (52).

Modern art's aspiration is uniqueness; progress has little or no meaning. The challenge for contemporary artists is not to extend an existing historical cultural line (that role has been handed off to craft), but rather to break from that line and create a territory not already occupied—a new form of expression that is not necessarily better but is different and distinct enough to be recognized as a new space by the art world (53).

Today, artists need a “logo,” a personal DNA, that they can own. Modern art's aspiration is uniqueness; disruption and progress have little or no meaning. Several modern practices may involve workforces of more than a hundred people with multiple skillsets, not only artists, but also others with skills in finance, marketing, and information technology. Such are the scales of the studios of artists such as Olafur Eliasson, Bjarke Ingels, and Anish Kapoor. Downsizing events in such practices make the news in today's art world, the scale of projects becoming enormous and art becoming intertwined with technology.

Contemporary art embodies mind-bending, challenging, thought-provoking, unclassifiable, and perplexing works, without any discernible mainstream and geographical centrality. There is a constant string of biennials (and some triennials), monumental exhibits, and amazing building investments in new museums by renowned architects. It is no longer possible to talk about the art world only in terms of the West. But for those receptive to new ideas and seeking to expand their horizons, an awareness of art's dizzying and divergent ways will undoubtedly enrich and expand the landscape of their science or engineering thinking. That is the value that modern art brings to the discussion.

## Creative fluidity breaks down distinctions among domains

Does combining modes of thinking lead to greater creativity? We assume that it does. In fact, that is presumably the idea of having universities on campuses rather that physically disconnected schools. I talked earlier of barriers to domain fluidity. Let us consider the benefits of bridging domains [some of what one sees in this space is eminently practical (54); that is not our interest here]. The observations can apply not only to individuals, but also to teams. In both cases, the key issue resides in understanding how others think.

To develop the framework, let us start by describing, as succinctly as possible, the modes of thinking that characterize science, technology, art, math, and humanities. As a first pass, technology goes with invention and science with discovery. Math goes with creation and, for the Platonists among mathematicians, also discovery. Applied math spans creation and invention. The science label is not an apt description of how computer science operates—but it is far too late to correct that term now. Nevertheless, computer science is a field of invention—as in developing new languages and technologies, artificial intelligence (AI) being a technology—as well as creation and even in discovery in the theoretical parts of the field that touch logic and pure math.

Art could be paired with creation, but most artists will pick another descriptor, e.g. express, reveal, provoke, incite, irritate, challenge, reframe, shock, upset, document, nauseate, etc. Creation is an outcome, a by-product. Some parts of engineering are indistinguishable from science, and discovery may be an apt descriptor, but most definitely building and invention go with engineering and, if design is part of the picture, so is creation. For completeness, let us associate humanities with interpretation and questioning; its goal is understanding, not creation or prediction. And, in some areas, like philosophy, process may be a better descriptor. Inquiry is part of a process but certainly not the objective or the goal, as discovery may be in science or invention may be in technology.

How can combining these modes of thinking break down domains and enhance creativity? For creative emergence, process is more important than focusing on outcome.

Skills and education may be incomplete descriptors of how someone may think. We need to think of people in broader terms and not immediately place them in a box connected with their training.

Many of us go through life looking at things through one pair of glasses, often very successfully. But having the possibility of adding another pair, understanding how others view questions and problems that may be quite different from our own enriches our thinking. Inspiration can emerge from domains outside our own.

I have commented before (55) about the Lunar Society, the Bauhaus, Black Mountain College, Bell Labs, and the Office of Scientific Research and Development as examples of organizations that produced remarkable amount of creative outputs. The *9 Evenings* event, the hugely successful collaboration between art and science that took place in October 1966 in the enormous space of the 69th Regiment Armory building in New York, is worth examining. Many current art-science discussions tend to focus on outcomes; finished pieces of art with some science motivation or content, pieces that can be placed in an exhibit. The *9 Evenings* project was different.

*9 Evenings: Theatre & Engineering*, as this was its complete title, brought together 10 artists (led by Robert Rauschenberg) and 30 engineers (led by Billy Klüver from Bell Labs) to create avant-garde pieces of theatre and dance. The two teams worked for 10 months to develop technical equipment and systems that were used as an integral part of ten artistic performances.

The culmination of this extraordinary, intensely ambitious collaboration produced many firsts. These included the use of closed-circuit television and television projection on stage, a fiber-optic camera that showed the audience objects in a performer's pocket, an infrared television camera that was able to record action in total darkness, a Doppler sonar device that translated movements into sound while wireless FM transmitters and amplifiers allowed speech, and a system where body sounds could be broadcast through loudspeakers.

This was a meeting of minds that went to the root of what makes for a meaningful art-technology-science connection: understand how the other side thinks.

In April 1995, Steve Jobs, in an oral history interview with Daniel Morrow for the Smithsonian, said: “I actually think there’s actually very little distinction between an artist and a scientist or engineer of the highest caliber.” This statement was made during a time when Jobs was leading NeXT, a company focused on pushing technological boundaries. A key point in this pronouncement is “highest caliber.” Jobs was not saying that all artists, engineers, and scientists are the same, but rather that the most exceptional individuals in each field share a similar mindset and skillset. This quote reflects Jobs's belief in the value of integrating artistic sensibilities into technical fields, which was evident went he went back to design-focused Apple. We all saw the value of this integration.

## Two broad domains: Where are we now?

Here, I will comment on two aspects of the broader creativity landscape. The first is the result of the combination of the availability of enormous amount of data with the ability to do analyses that were before considered unthinkable. The second one can be phrased as “Can we create creativity?” As one may imagine, AI will appear in both sets.

What are characteristics of creative output of teams that can be studied in a longitudinal way? Let us first present a few results and areas that have been opened to computational inspection.

A work that opened this line of thinking focused on Broadway musicals (56). This was an inspired choice; the creative output of Broadway teams has been documented since the very first production a century and half ago. We know who contributed to the creation of a play and how successful the play was. The size of the teams is small and typically includes a composer, a librettist, a lyricist, a choreographer, a director who manages the team's collaboration, and a producer who manages the financial backing. What is the combination of experienced members and newcomers that produce the most successful team? Do teams need the infusion of new members to maintain success? Those were the questions tackled by this first study. Guimerà et al. (57). followed up with how teams self-assemble and studied collaboration networks in artistic and scientific fields.

Other studies examined increasingly larger datasets expanding studies to science and engineering. Wuchty et al. (58) studied 19.9 million papers and 2.1 million patents covering engineering, social sciences, arts, and humanities to demonstrate that teams increasingly dominate over solo authors in the production of knowledge. In another study, Uzzi et al. (59) examined 17.9 million papers, spanning all scientific fields, focusing on the balance of atypical knowledge with conventional knowledge and its effect on impact. Sinatra et al. (60) studied the evolution of impact and productivity of thousands of scientific careers. Liu et al. (61) studied “hot streaks”—periods during which an individual's performance is substantially better than their typical performance—in the career histories of individual artists, film directors, and scientists, examining artworks, films, and scientific publications. Wu et al. (62), in a study involving the production of more than 65 million papers, patents, and software products, showed that smaller teams tended to disrupt science and technology with new ideas, whereas larger teams have tended to expand and develop existing ones.

Growth in this space has been fast. The “manifesto” of the many authors involved in Fortunato et al. (63), announcing “Science of Science” as a new discipline—the area that focuses on the use of data analysis, complexity, and AI to understand the patterns and mechanisms of scientific breakthroughs and innovative developments—has already come to pass (64).

Let us go now to the second topic: can we generate creativity? Or can we do “creativity without magic”?

“There is now a considerable body of evidence that problem solving of a kind that would be regarded as creative if exhibited by human beings can be produced by computer programs,” scholar Herb Simon said in a 1983 paper. Prior to that, in 1958, Simon and Allen Newell wrote (65), “within ten years a digital computer will be the world's chess champion.” When it did not happen, he was widely ridiculed (as was the whole field of AI). Of course, Deep Blue beat Kasparov in 1997. So, it took 39 years, not 10 years.

But in the intro to that 1983 paper (66), Simon described the characteristics of creative individuals in term of 3 characteristics: “(i) willingness to accept vaguely defined problem statements and gradually to structure them, (ii) continuing preoccupation with problems over considerable periods of time, and (iii) extensive background knowledge in relevant and potentially relevant areas.” He went on to say that “not all of these conditions tolerance of ambiguity, persistence, and knowledge are satisfied in all cases of discovery, but their presence has been observed and commented on too many times to suppose that their association with success in discovery is accidental.” But in the second part of the paper, he goes into computer models to rediscover knowledge. “These programs have been tested mainly not by setting them out to search for genuinely new knowledge … but by setting them the problem of rediscovering important scientific laws and concepts, starting from essentially the same situations that the original human discoverers did.” None of the models he described look like what AI looks like in today's large language models. But AI was already there. By 1980, we had *AI Magazine*, now under the umbrella of The Association for the Advancement of Artificial Intelligence. *AI Magazine* has been publishing continuously since 1980.

By 1995, Simon's references to AI appear loud and clear. “Artificial intelligence methods may be used to model human intelligence or to build intelligent (expert) computer systems. AI has already reached the stage of human simulation where it can model such “ineffable’ phenomena as intuition, insight and inspiration…It is now just forty years since Al Newell, Cliff Shaw and I took the plunge into the exhilarating waters of AI. We called it “complex information processing” (67). AI, and the questions it has generated, has been around for a long time, but I am not sure if many would be able to defend the claim that AI has already reached the stage of human simulation in which it can model such “ineffable” phenomena as intuition, insight, and inspiration.

By 2009, views were decidedly positive. “There are three forms: combinational, exploratory, and transformational. All three can be modeled by AI-in some cases, with impressive results,” proclaimed Margaret Boden in AI Magazine (68).

## AI creativity is its own burgeoning field

What is AI capable of now? In terms of creativity, AI excels at combinatorial creativity. It can easily combine old ideas together to create something. But transformational creativity remains a challenge. Could AI create break-withs, produce the kinds of conceptual advances that would make scientific news?

There are reasons to be optimistic. The protein folding problem was considered extremely hard until 2020, when AlphaFold, an AI program developed by developed by Google DeepMind, predicted, with a high degree of accuracy, the 3-dimensional structure of proteins based solely on its amino acid sequence. But whereas protein folding is example of the prowess of AI, it may not be good example of creativity associated with AI. This may fall as an example of the kinds of problem solved by deep learning.

But the praise and dangers surrounding AI today makes it hard to think clearly—at least in its current form—about what AI can and cannot do. In fact, it has been argued that defining AI as “the ability to accomplish complex goals,” is appropriate for machines but falls short of capturing the essence of human thought (69).

There are areas of clear overreach. One such area is in the arts. AI-assisted illustration is fine (70), though there are dangers when the tools exceed our imagination and judgment (71). But asking art historians, critics, and gallerists if an AI-generated painting could have been done by abstract painter Mark Rothko misses the point in exploration of creativity. Rothko happened, and that is the end of it. There is no space for a second Rothko in the history of art.

As a creative aid, there is no doubt that AI-driven tools will be embraced by some artists and ignored by many others. Many painters, writers, and philosophers (72) recoiled at the invention of the camera, which they saw as a defilement of human artistry. The French poet Charles Baudelaire called photography “art's most mortal enemy.” But Man Ray, the American-born, French-based artist, embraced photography in the mid-1920s and produced amazing work. Photography ultimately became recognized as art. Whether AI follows the same path remains to be seen.

As to break-withs, let us consider a concrete thought experiment. Pretend it is 1970, and AI as we know it already exists. You are an architect based in Los Angeles who designs hotels with big atriums. You want new ideas, and you ask AI for help. AI knows everything that has happened with buildings in Los Angeles, the United States, and the world within the past 200 years. If you ask AI to give you 100 ideas, could any of those anticipate what John Portman did in the mid-1970s, making elevators visible in atriums or putting them outside the building? Most likely no, since no pre-Portman building had used this idea. Perhaps AI could have come up with it if you instructed it to “put the elevators anywhere.” But then, of course, the question already contains the answer.

I would argue that any machine analysis of the entire history of physics prior to 1900 would not have been able to come up with quantum mechanics or relativity. Why? Because the new way of thinking would violate the very cornerstone of prior knowledge; every piece of knowledge AI would have access to contains this as an assumption. Could AI have known that energy was discontinuous or that space and time were equivalent by looking backward? In the same pre-1900 time frame, could AI have come up with cubism? It is doubtful; all Western art prior to 1900 was representational. Generative AI is entirely based on the past, so it cannot break from the past to create fundamental innovations. Transformational creativity is rare and infrequent, so it is tempting to equate with the essence of human genius. In 1968, in what was a different era, especially in computer-years, Picasso said, “Computers are useless, they can only give you answers.” It’s a bit extreme, but he had a point. Questions are essential if we want AI to produce break-withs.

It is still unclear what kind of questions will unlock barriers and open new vistas. Could it be possible that if we ask, “How can we solve the fusion problem?” or “What is the technology that will make quantum computing practical?” that AI would magically give us a solution? What you will get now is a summary of where things are.

Another example, less ambitious perhaps, but that brings the issue of what could have been the question that would have open this area and jumped started this area 40 or 50 years ago (assume that AI had existed then). Origami, the Japanese art of paper folding, has been around for at least 500 years. But at some point, in the last several decades, it became of interest to mathematicians and engineers. So much has happened in this space that now we have an entire domain called origami engineering. Applications range from packaging to architecture, robotics, medicine, and aerospace. Being able to transport something small—the critical issue in transporting things to space—and having it unfold to produce a solar panel half the size of a tennis court is something that would have been unthinkable in 1960. Now, it looks easy. What kinds of questions could have generated this merger of ideas? A question such as “Can you find applications of origami to solve issues in engineering” already contains the answer.

How can we ask questions to find unexploited territories? Questions generating those types of rich connections rarely emerge as epiphanies, fully formed prescriptions on how to develop an entire new area. Either way, the path to using it as our creative assistant lies in learning to ask better questions to unlock creative potential. It may well be that asking good questions is the essence of creativity. Perhaps generative AI will get there, but for now, on the break-with side, humans seem to hold the upper hand.

## Data Availability

All data are included in the manuscript.
